# Influence of Calcium, Magnesium, and Iron Ions on Aerobic Granulation

**DOI:** 10.1007/s12010-014-1236-0

**Published:** 2014-09-21

**Authors:** Beata Kończak, Jagna Karcz, Korneliusz Miksch

**Affiliations:** 1 Environmental Biotechnology Department, Faculty of Power and Environmental Engineering, Silesian University of Technology, Akademicka 2, Gliwice, Poland; 2 Laboratory of Electron Scanning Microscopy, Faculty of Biology and Environmental Protection, Silesian University, Katowice, Poland

**Keywords:** Aerobic granule, Multivalent cations, Formation, Stability

## Abstract

In this study, we investigated the effect of different multivalent cations on granule formation. Previous experiments showed that formation of matrix EPS and their structure depend of the presence of divalent cations. This study indicates that trivalent cations are also playing an important role. However, the more compact granules were formed in the presence of all cations. The authors tried also to determine changes in proteomic profile of slime and tightly bound EPS. These results showed that matrix EPS is composed of a variety of large and complex proteins, but there are also small proteins, like for example, lectins. These small proteins have a role in the interaction between cells and exopolysaccharides and in granules formation.

## Introduction

The aerobic granular system is a promising technology for compact wastewater treatment plants. This system is superior to conventional activated sludge processes, in terms of high biomass retention, high conversion capacity, less biomass production, excellent settleabilty, and resistance to inhibitory and toxic compounds [1–5]. Some of the requirements for the formation of aerobic granules are as follows: specific substrate composition, feast-famine regime, relatively high hydrodynamic shear force, and short settling time [6, 7].

Extracellular polymeric substances (EPS), like exoprotein, exopolysaccharides, or extracellular DNA (eDNA), have a positive effect on aerobic granulation [8, 9]. Besides, the formation of granules is possible only in the presence of divalent cations. EPS, by means of presence of divalent cations, play a cementing role in connecting individual cells into the three-dimensional structure of an aerobic granule [10]. A partial neutralization of negative charges in bacterial cell surfaces by divalent cations could stimulate granulation [11, 12]. Li et al. [13] noted that augmentation with divalent cations significantly decreased the sludge granulation time from 32 to 18 days. His results showed that in the presence of divalent cations, aerobic granules had more compact and dense structure. These investigations help to understand the complex interaction between EPS and metal ions but there is still a lack of knowledge about the different effects of Ca^2+^, Mg^2+^, and also Fe^3+^ on aerobic granulation and granules performances.

Park [14] noted that Fe^3+^ has higher flocculating ability than divalent cations, building strong and compact flocs. According to Schultz-Hardy rule [15], the coagulation capacity depends upon the valence of the active ion carrying charge opposite to the charge on the colloidal particles. Coagulating power of an electrolyte is directly proportional to the valence of these active ions. The coagulation power of different cations has been found to decrease as: Al^3+^ > Mg^2+^ or Ca^2+^ > Na^+^ [16–18]. The main objective of this study was to gain a comprehensive view of the interactions between EPS and three kinds of multivalent metal ions. It is expected that the information provided here would be useful to further understand the mechanism of aerobic granules formation and facilitate the application of aerobic granulation technology in wastewater treatment.

## Material and Methods

### Reactor Setup and Operation

Six bubble columns (SBR A to F) with a working volume of 1 L each were used to cultivate aerobic granules. The internal diameter of the reactors was 40.5 mm, and the reactors working *H*/*D* ratio was about 6.9. Fine air bubbles for aeration were supplied through an air diffuser at the reactors bottom.

The reactors were inoculated with activated sludge obtained from a local municipal wastewater treatment plant in Zabrze, Poland. The reactors were operated in 3-h cycles with 3-min filling, 5-min settling, 3 min of discharge, and 1 min of idle period. This led to 168 min of aeration in one cycle at steady-state operation. Effluent was discharged from the middle port of the reactors with a volumetric exchange ratio of 50 %. Hydraulic retention time was 6 h and organic loading rates were about 2.4 kg COD/(m^3^ days).

### Medium

A synthetic wastewater with the following compounds was used in the experiments (g/L): COD (sodium acetate) 0.543, (NH_4_)_2_SO_4_ 0.075, and KH_2_PO_4_ 0.075. A trace solution was added to the feeding media (g/L): H_3_BO_3_ 0.05, ZnCl_2_ 0.05, CuCl_2_ 0.03, MnSO_4_ · H_2_O 0.05, (NH_4_)_6_Mo_7_O_24_ · 4H_2_O 0.05, AlCl_3_ 0.05, CoCl_2_ · 6H_2_O 0.05, and NiCl_2_ 0.05. Table 1 presents the augmentation strategy with addition of Ca^2+^, Fe^3+^, and Mg^2+^ to the reactors. pH of feeding media was fixed at 7.2.

**Table 1:** Scheme of the multivalent cation addition into reactors

		SBR A	SBR B	SBR C	SBR D	SBR E	SBR F
Concentration (mmoL/L)	Mg^2+^	–	–	0.125	0.125	–	0.125
	Fe^3+^	–	–	–	–	0.075	0.075
	Ca^2+^	–	0.125	–	0.125	–	0.125

### Extraction of EPS

Sludge fractioning protocol was modified according to the method previously described by Yu et al. [19]. In brief, the sludge sediments were centrifuged at 2000*g* for 15 min. The samples were centrifuged at 20,000 r/min, 4 °C for 20 min, and the supernatants were filtered through a 0.45-μm membrane of cellulose acetate. The supernatans were removed and the collected bottom sediments were re-suspended to their original volumes using a 0.1-M PBS (pH 7.4) buffer solution. Then, TB–EPS (tightly bound extracellular polymeric substances) were extracted using a cation exchange resin (Dowex 50 × 8, Fluka, USA; at 4 °C for 1 h). The dose of Dowex resin was 60 g/g VSS. The samples were centrifuged at 20,000 r/min, 4 °C for 20 min, and the supernatants were filtered through a 0.45-μm membrane of cellulose acetate. The filtrates were used as TB– EPS samples.

### Chemical Analysis

Sludge from batch reactors was sampled weekly to measure the protein (PN), carbohydrate (PS), sludge volume index (SVI), total suspended solids (TSS), and volatile suspended solids (VSS) concentrations in duplicate.

TSS and VSS were analyzed in accordance to the standard methods [20]. The carbohydrate content (PS) of EPS was measured by the phenol-sulphuric method using glucose as the standard [21]. The content of protein (PN) in the EPS was measured using fluorescent dyes binding to protein (Quant-IT, Invitrogen). The fluorescence intensity of the resulting complex depends directly on the amount of protein in the sample.

## Microscopy Techniques

### Image Analysis

Changes in morphology of the granules were followed by image analysis. Images of the granular sludge were taken with a camera Moticam BA400 combined with a microscope Motic 245A. For digital image analysis the program Image Plus was used. The diameter was calculated as an average value from the shortest and the longest measured segment in the granule.

### Scanning Electron Microscopy

Granule samples were removed from the growth medium in the reactor and washed in a 0.1-M phosphate buffer solution (pH 7.4). The samples were then lyophilized for 4 h using Christ Alpha 4 L Lyophilizator, operated in the temperature of −50 °C and the pressure of 4 Pa. After complete dehydratation, samples were attached onto stubs with double-sided adhesive (carbon type) and sputter-coated in a Pelco SC-6 Sputter Coater with a thin film of gold to improve electrical conductivity of the sample surface. The samples were imaged by the Tesla BS 340 scanning electron microscope in high-vacuum mode operating at 20 kV with secondary electron detector (ESD), and working distance (WD) of 10–30 mm. Digital photomicrographs were taken using Microsystem SEM Framegrabber V 1.1.

### Statistical Analyses

All measurements were carried out in duplicate. The data points in the figures show the average and the error bars show one standard deviation. The *t* test was used to determine the *p* values and statistical significance (*p* < 0.05) of results.

## Results

### Biomass Concentration and Formation of Granules

**Fig. 1:**
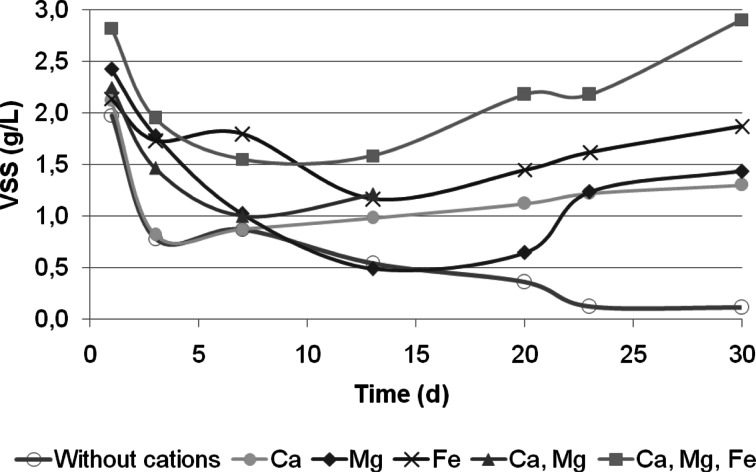
Evolution of biomass concentration

The sludge used in all systems as inoculum was the typical flocculent activated sludge with a fluffy, irregular, and loose morphology and relative abundance of filamentous microorganisms. After the SBR was started up, the concentration of biomass was decreased because an important amount of suspended biomass was washed-out from the reactor (Fig. 1). This was a result of the operation strategy of the systems, in which a very short settling time and a fast effluent withdrawal period were applied to all reactors.

**Fig. 2:**
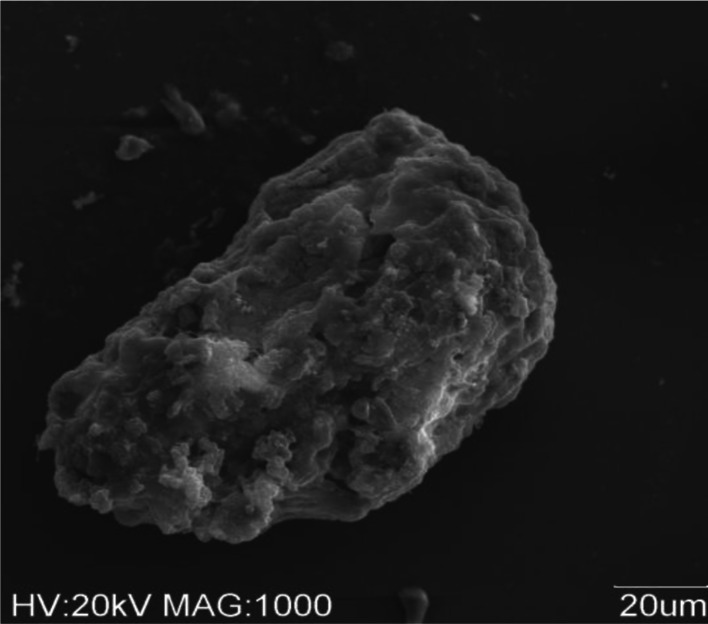
SEM image showing the surface topography of an entire Ca^2+^-fed granule

Compared with aerobic granules cultivated without cation addition, the cation-fed granules had more rigid structure. SEM observation showed the granules with high degree of compactness (Fig. 2). In these reactors, a faster granulation process was observed and the start-up period was shorter. Especially, in the presence of iron cations, the biomass retention increased (Fig. 1). From day 13, the formation of small, roughly aggregates with an average diameter of 1.15 mm was observed in all the reactors with cation addition.

However, only in the Ca^2+^-Mg^2+^-Fe^3+^-fed reactor, suspended flocs gradually disappeared from the reactor. In this reactor, from day 23, an intense growth of biomass was observed. On the contrary, in the control reactor without addition of cations, the biomass was almost completely washed-out (Fig. 1).

### Secretion of TB–EPS

**Fig. 3:**
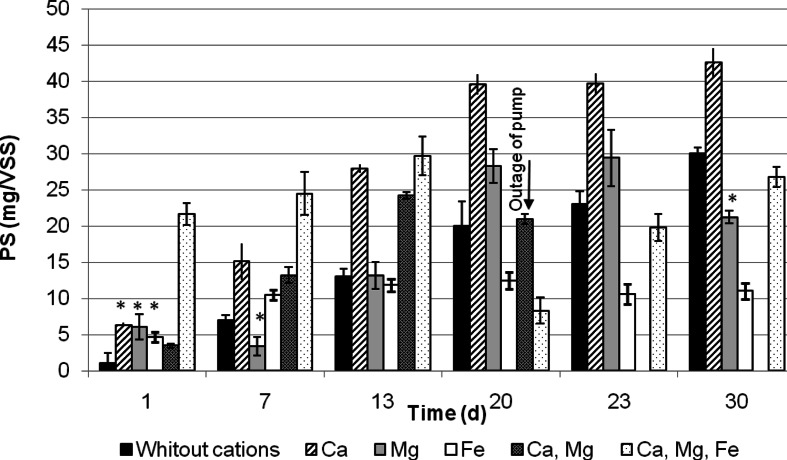
Evolution of polysaccharides content in TB-EPS during granules formation. (*p < 0.05, not statistically significant results)

**Fig. 4:**
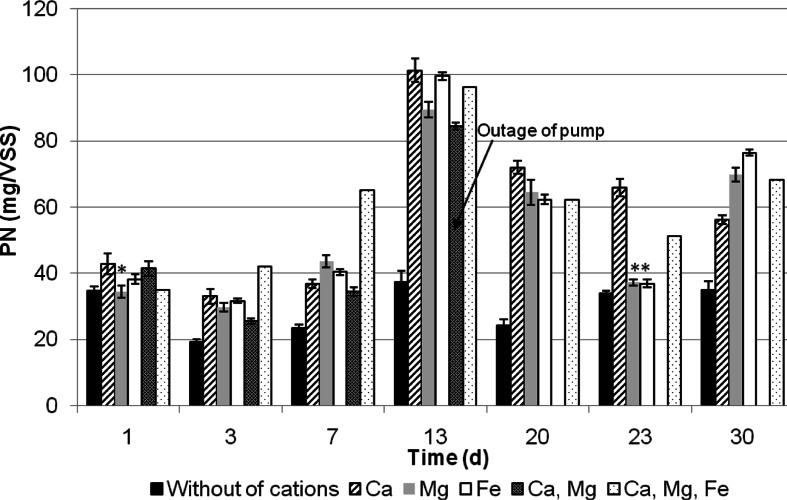
Evolution of protein content in TB - EPS during granules formation. (*p < 0.05 not statistically significant results)

Figures 3 and 4 show the evolution of TB–EPS during granulation. It was observed that divalent cations play a role in the binding of polysaccharides. Secretion of polysaccharides in the SBR A without addition of cations was insignificant. Most intense secretion of polysaccharides was observed in the presence of Ca^2+^; moreover, in the Ca^2+^-fed reactors, degradation of polysaccharides was very low. Until day 14, the increase of polysaccharides secretion was noted also in Ca^2+^-Mg^2+-^Fe^3+^-reactor; hereupon, the content of polysaccharides decreased slowly and began to stabilize. The presence of Mg^2+^ had also an influence on the secretion of polysaccharides, while the presence of Fe^3+^ was not important for polysaccharides exude. In the SBR D with the Ca^2+^ and Mg^2+^ addition, synergistic effect was not observed, if anything it was observed that the content of polysaccharides decreased. Outage of a pump after 14 days of operation disabled the continuation of research in this reactor (Fig. 3).

The divalent and mutivalent cations play a role in the process of exoproteins binding. The lowest concentration of protein was observed in the SBR A without cation addition. Most intense secretion of proteins was observed in the presence of Ca^2+^ and Fe^3+^ (Fig. 4). It could be also observed that until the 13th day of granulation, the protein production increases. Then, slowly decreases and more or less stabilized.

### Structure of Granules

**Fig. 5:**
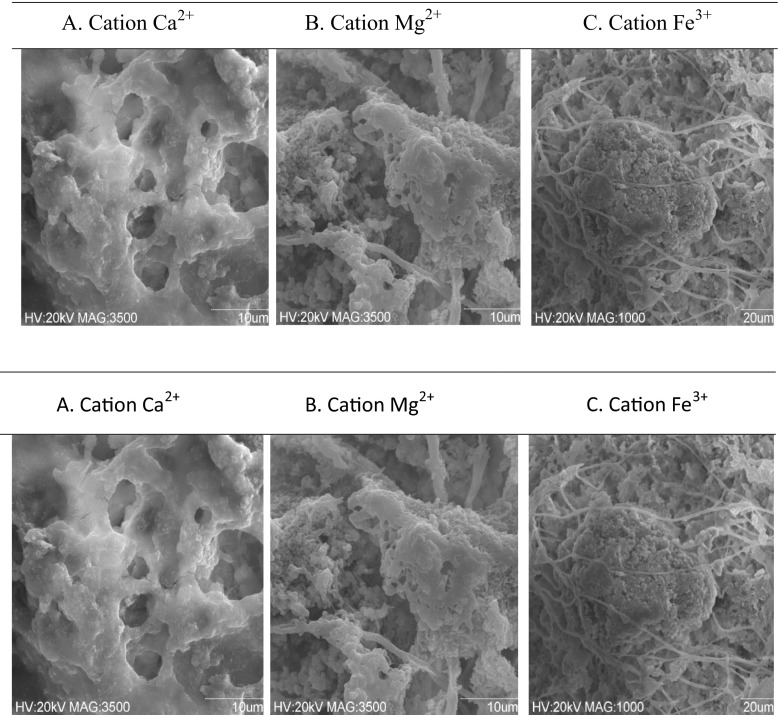
Selected SEM images of the EPS matrix in the presence of: **a** Ca^2+^, **b** Mg^2+^, and **c** Fe^3+^

Scanning electron microscopy (SEM) showed a three-dimensional structure of the EPS matrix which developed in the granular sludge. Figure 5a shows that in the presence of Ca^2+^, the matrix structure of EPS was homogeneous and had a hydrogel-like form. SEM observation showed also that a part of EPS build the skeleton of granules and bacteria were attached to this skeleton. Aerobic granules were also composed of pores and channels that were not plugged by EPS and which enabled mass and oxygen transfer into granules.

In the presence of Mg^2+^, the EPS matrix structure was heterogeneous. Compared to Ca^2+^-fed granules, the extracellular matrix was formed of an enveloping layer and fibrous material which linked each cell to cell. Probably, it was filamentous protein (Fig. 5b).

Previous results showed that Fe^3+^-fed granules had a dense structure, what protected them from washout.

The structure of Fe^3+^-fed granules was very heterogeneous. Figure 5c shows that in the presence of Fe^3+^, dark aggregates with a large amount of bacteria grew up. These aggregates had entwined fibrous material, providing stability and high granular density. On the other side, in the EPS network, there were many pores and channels, which enabled the mass and oxygen transport through the granules.

## Discussion

From the 1st day of granulation, the production of EPS, especially polysaccharides, is very intense. Regardless of the reactor, the scheme of protein (PN) secretion was almost the same. Until the formation of first granules is finished, the concentration of PN increases. Then, the protein production decreases, and the excess of EPS is biodegradable. Gao et al. [22] also reported that EPS could be utilized by the bacteria themselves, since at least 50 % of the polysaccharide (PS) and 30 % of the protein (PN) in the EPS produced by aerobic granules are biodegradable.

The results showed that TB–EPS play a significant role in the formation of granules, which is in accordance with the results of Chen et al. [23].

The structure of Ca^2+^-fed granule had a hydrogel-like form. According to Sankalia et al. [24], the formation of hydrogel is the result of the interaction between Ca^2+^ and alginate-like polysaccharides. Lin et al. [25] showed that the entrapment of cells in alginates cross-linked with Ca^2+^ has great potential in biofilm and granules formation. Although, the structure of hydrogels is relatively dense, they have many pores with diameters on the micrometer to millimeter scale. As a consequence, substrate and oxygen are able to freely diffuse into granules [26].

Similar results were obtained for Mg^2+^-fed granules. However, in the presence of Mg^2+^ beside of hydrogel-like matrix, fibril material was also formed. But in general, the granule structure was similar. Ensue from this, the mechanism of granules formation in the presence of both cations is almost the same.

Interestingly, in the reactor with Ca^2+^ and Mg^2+^ augmentation, the synergistic effect was not observed. On the contrary, the polysaccharide production decreased. Mg^2+^ can cause ion exchange with Ca^2+^ inside the granule, making more difficult the interaction between Ca^2+^ and polysaccharides.

Concurrent augmentation with Ca^2+^ and Mg^2+^ reduced the specificity of granules, which is a result of the character of the formed matrix. Hydrogels can adsorb a large amount of water, but this influences negatively the granule settling velocity. Differently, the augmentation with Ca^2+^, Mg ^2+^, and Fe^3+^ together benefits the granulation. Because of that, it can be hypothesized that Fe^3+^ plays an important role in biogranulation. In the presence of Fe^3+^, the process of biomass washout decreases. Similarly to divalent cations, Fe^3+^ may bind EPS, but in addition, it participates in bio-coagulation. Because of that, organic and inorganic molecules may be adsorbed on the surface of Fe^3+^-fed granules. Fe^3+^-fed granules consisted of many compact aggregates, binding together by EPS and fibril material. For this reason, the specific weight of granules increased and they can be retained inside the reactor.

## Conclusion

Analyzing the significance of cations and EPS on the mechanisms of granulation, it can be determined that the most important processes are the formation of a three-dimensional polysaccharide network with embedded EPS and cells to support the stability of granules. In the presence of divalent cations (Ca^2+^ or Mg^2+^), the polysaccharide network was well developed. However, only in the presence of all cations (Ca^2+^, Mg^2^, Fe^3+^), granules have the most compact structure and they are stable. Besides, this type of granules is characterized by good settling properties, which minimized biomass washout from the reactor.

It was found that divalent and trivalent metal ions show different effects on the granulation process and also led to the different characteristics of mature granules in SBRs.

The results in this study demonstrated that the mechanism of granule formation is complicated. There are many factors apart from shear forces and short settling time which influence granule formation. The results obtained may be useful information for the future researches.
